# An Eco-Friendly Polymer Composite Fertilizer for Soil Fixation, Slope Stability, and Erosion Control

**DOI:** 10.3390/polym14071461

**Published:** 2022-04-03

**Authors:** Tao Li, Fengli Dai, Yufeng He, Daqian Xu, Rongmin Wang

**Affiliations:** 1 School of Chemical Engineering, Lanzhou City University, Lanzhou 730070, China; daqianxu@126.com; 2 Institute of Polymer, College of Chemistry and Chemical Engineering, Northwest Normal University, Lanzhou 730070, China; daifl99@126.com (F.D.); heyufeng@nwnu.edu.cn (Y.H.)

**Keywords:** polymer composite fertilizer, corn straw wastes, soil fixation, slope stability, environmental remediation

## Abstract

In the Loess Plateau region, the poor structure and properties of loess slopes will cause many types of geological disasters such as landslides, mudflow, land collapse, soil erosion, and ground cracking. In this paper, an eco-friendly polymer composite fertilizer (PCF) based on corn straw wastes (CS) and geopolymer synthesized from loess was studied. The characterization by FT-IR of the PCF confirmed that graft copolymer is formed, while morphological analysis by scanning electron microscopy and energy dispersive spectroscopy showed that geopolymer and urea were embedded in the polymer porous network. The effects of PCF contents on the compressive strength of loess were investigated. The PCF was characterized in terms of surface curing test, temperature and freeze-thaw aging property, water and wind erosion resistance, and remediation soil acidity and alkalinity property, which indicates that PCF can improve loess slope fixation and stability by physical and chemical effects. Moreover, the loess slope planting experiment showed that PCF can significantly increase the germination rate of vegetation from 31% to 68% and promote the survival rate of slope vegetation from 45.2% to 67.7% to enhance biological protection for loess slopes. The PCF meets the demands of building and roadbed slope protection and water-soil conservation in arid and semi-arid regions, which opens a new application field for multifunctional polymer composite fertilizers with low cost and environmental remediation.

## 1. Introduction

The Loess Plateau is located in the middle and upper reaches of the Yellow River in Northwest China and covers a total area of about 648,700 km^2^. Loess soil is a typical type of aeolian silt, mainly formed by the sedimentation of wind-blown dust, which is sensitive to wind and water erosion [1,2]. Loess slopes have become extremely unstable due to the increasingly fragile environment and highly fragmented and complex terrain, steep slopes, and poor vegetation condition [3,4], and it leads to surface erosion of loess slopes and may even cause severe geological disasters, such as soil erosion, even mudslides and landslides [5]. In addition, the cities in Northwest China, such as Lanzhou City, have been accelerating urbanization to boost their economies and improve living standards [6], and human activities, such as transportation construction, agricultural irrigation, residential extension, and environmental depredation seriously decrease the stability of loess slopes [7,8] and result in soil erosion, which produces on-site soil and nutrient loss, vegetation cover reduction, and land degradation [9]. 

At present, loess slope protection technologies are mainly focused on theoretical studies, such as slope stability mechanisms [10], water-soil erosion control [11], biological protection by vegetation [12], and the root–soil system model [13]. In terms of practical materials and technology, various traditional treatment methods for loess slope protection (e.g., anti-slide piles, geogrid, and retaining walls) have been widely applied to improve soil strength and stability [14,15] and reduce soil erosion [16]. However, these treatments can not completely address water and soil erosion problems and achieve the long-term stability of loess slopes. In addition, the widespread application of traditional reinforcement materials, especially polymer materials [17,18,19], is limited largely due to their large size, which leads to uneven mixing with soil. With the approval of green and sustainable conceptions, vegetation stability for slopes has been widely proven an effective, green, and long-term approach to improve slope fixation and control water-soil erosion, especially in arid and semi-arid regions [20]. However, large-scale vegetation protection is restricted by soil structure and fertility, rainfall, the survival rate of seeds, and the growth of roots [21,22]. Therefore, reasonable design and development of eco-friendly polymer materials that combines physical, chemical, and biological protection properties is a key field in effective, green, and long-term loess slope stability and water-soil erosion control [23].

In this paper, an eco-friendly polymer composite fertilizer in combination with loess-based geopolymer, corn straw, and acrylic acid (AA) was developed, which offers several advantages: good heat resistance, washing resistance, freeze-thaw resistance, wind erosion resistance, and surface curing properties. In addition, it can regulate the pH value of soil, improve the germination rate and promote the growth of loess slope vegetation. This material combines physical, chemical, and biological technology to protect artificial and natural loess slopes by reducing slope water-soil erosion damage and enhancing comprehensive stability. 

## 2. Materials and Methods

### 2.1. Materials

Loess (The organic matter content is 0.3–0.8%) and corn straw were collected from Lanzhou City (China). Acrylic acid (AA) was provided by Shanghai Maclean Biochemical Company (Shanghai, China). N, N-methylene bisacrylamide (MBA) was supplied from Chengdu Chemical Reagent Factory (Chengdu, China). Urea was purchased from Beichen Founder Reagent Factory (Tianjin, China). All the chemicals used were analytical reagent grade, and deionized water was used in the preparation of the eco-friendly polymer composite fertilizer (PCF).

### 2.2. Preparation of PCF

An amount of 3 g loess-based geopolymer by alkali-activated hydrothermal reaction [24], 10 g urea, 1.5 g corn straw powder, and 35 mL distilled water were added into a 250 mL three-neck flask with magnetic stirring under nitrogen atmosphere. The mixture was stirred vigorously for 45 min and heated to 75 °C. Then, 0.1 g potassium persulfate solution (KPS) and 7.2 g AA (neutralization degree of 60%) were slowly added and stirred for 2 h. An amount of 0.3 g MBA was added and stirred continuously under nitrogen atmosphere for 30 min. Afterward, the product was removed, washed with ethyl alcohol three times, and dried to constant weight at 60 °C. Finally, the product was crushed to 100 mesh and PCF was obtained. 

### 2.3. Methods of Characterization 

The structures of the PCF were characterized using an infrared spectra spectrometric analyzer (FT-IR, FTS3000, DigiLAB Merlin, Holliston, MA, USA) at 400–4000 cm^−1^. The surface morphology and the surface elemental distribution of PCF were examined using scanning electron microscopy and energy dispersive spectroscopy (SEM and EDS, ULTRA PLUS, Zeiss, Oberkochen, Germany) after coating the samples with gold film.

### 2.4. Compressive Strength Property

**Figure 1:**
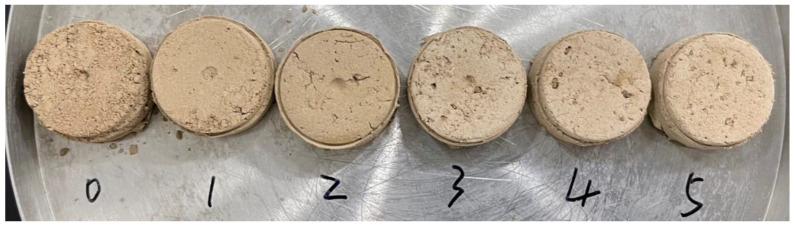
The appearance of the loess columns containing 0%, 1%, 2%, 3%, 4%, and 5% PCF (based on loess weight), respectively.

Firstly, the loess column samples (1.50 cm in height and 2.65 cm in diameter) were prepared according to the previous study [25]. In detail, the soil columns mixed with different amounts of PCF were prepared as follows: a certain amount of loess, PCF (0%, 1%, 2%, 3%, 4%, and 5%, based on loess weight), and 5 mL tap water were mixed well. The mixture was put into a plastic mold and pressed tightly. Then, these plastic molds with samples were dried at room temperature for 24 h until the compact crumb structure was formed. The appearance of the loess columns containing different amounts of PCF (based on loess weight) is shown in Figure 1. Measurement was carried out using a press machine (Cangzhou JiluTest Instrument Co., Cangzhou, China) until columns cracked, and the value was recorded as the compressive strength.

### 2.5. Remediation Soil Acidity and Alkalinity by PCF

Firstly, simulated loess solution was obtained by centrifugation: 200 g dry loess was soaked in 1 L distilled water for 24 h and centrifuged under 1000 rpm for 3 min, and the supernatant was obtained as simulated soil solution. The influence of PCF on pH values of soil was measured by different amounts of PCF in simulated soil solution. After the swollen PCF was filtered, the pH value of the filtrate was measured by a pH meter.

Then, the pH values of the simulated soil solution were adjusted with 0.1 mol/L HCl or NaOH aqueous solution. An amount of 0.1 g of PCF samples was immersed in 50 mL of soil solution with different pH values at room temperature. After the swollen PCF was removed, the pH value of the filtrate was determined by a pH meter.

### 2.6. Temperature of the Aging Property

In the thermal test, the loess column samples were exposed to an air-dry oven at 45 °C for 24 days. The compressive strength changes of the loess column samples were measured after different aging times, and the tests were carried out continuously and without interruption in the whole process.

### 2.7. Freeze-Thaw Resistance Performance

In a freeze-thaw cycle, the loess column samples were frozen at −15 °C for 2 h and thawed at 25 °C for 2 h, alternately, and the freeze-thaw test contained seven cycles. The compressive strength of the loess column samples was measured after different freeze-thaw cycle times.

### 2.8. Surface Curing Test

Firstly, a simulated loess slope was constructed as the following method: 10 kg loess (<300 mesh, from Lanzhou South Mountain, Lanzhou City, China) was piled up a simulated loess slope (33 cm length, 15 cm width, 15 cm height, and 45° gradient). An amount of 100 g loess and 5 wt% PCF were mixed and spread evenly on the simulated loess slope surface, and the blank group without PCF was compared as control under the same condition. Then, 350 mL tap water was sprayed on the slope surface and placed in an outdoor environment, and the surface curing of different loess slopes was observed and photographed after 60 days. Meanwhile, the content of aggregate topsoil was determined by the dry-sieving method [26] as follows: 25 cm^2^ topsoil of loess slope was removed, weighed (marked as W_0_), and sieved (<0.25 mm). Then, the remaining topsoil was weighed again (marked as W_1_), and the content of aggregate soil ratio (COAs%) of the loess slope was calculated from the below equation:

(1) $$\mathrm{COAs}\%=\frac{W_{1}}{W_{0}}\times 100\%$$

### 2.9. Washing Resistance Test

10 L/m^2^ tap water was sprayed on a simulated loess slope treated with PCF, and another simulated loess slope without PCF was compared as control under the same amount of tap water. Then, photos were obtained from the water permeability of the simulated loess slope. In addition, the cover area and mass of loess washed down from the simulated loess slope were calculated and weighted after natural drying.

### 2.10. Wind Erosion-Resistance Test

Simulated loess slopes with different PCF contents (from 0 wt% to 5 wt%) were blown by simulating natural wind at a speed of 36 kilometers per hour (similar to level 6 of wind force), and the mass loss of slope topsoil was weighted and calculated before and after blowing.

### 2.11. Degradation Behavior of PCF in Soil

To examine the degradation behavior of the PCF, the dry weight loss of PCF was determined. Then, PCF was incubated in loess at a regular time interval, and then the samples were obtained, dried, and weighed. The ambient temperature and the soil humidity were maintained at room temperature and about 30% during the degradation experiment, and the degradation behavior was evaluated by weight change [27]. 

### 2.12. Influence of PCF on Seed Germination

In order to study the effect of PCF on the germination rate of vegetation, *Cyperaceae* for greening and biological slope fixation were chosen and cultivated at a density of 10 seeds per 25 cm^2^. A cultivated soil without PCF was compared as a control under the same condition. Then, the same amount of tap water was sprayed every day, and the germination of seeds was observed after a week of incubation.

### 2.13. Slope Planting Experiment

In brief, 100 g loess and 5 wt% PCF were mixed and spread on the surface of a simulated loess slope. A blank group without PCF was used as a control group. Afterward, 400 seeds of *Cyperaceae* were sown on simulated loess slopes, respectively and two simulated loess slopes were placed at room temperature. After germination, 100 mL water was sprayed every 2 days, and the germination of seeds and cracks on the slopes were observed and recorded after two months of incubation.

## 3. Results and Discussion

### 3.1. Preparation of PCF

**Figure 2:**
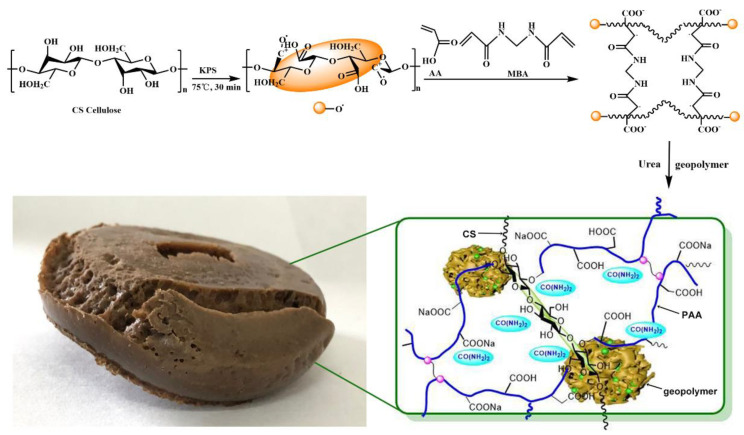
The reaction mechanism, chemical structure, and photograph of PCF.

The eco-friendly polymer composite fertilizer was prepared by graft polymerization and composite. CS was grafted with polyacrylic acid (PAA), and MBA and urea-loaded geopolymer were blended to form polymer composite gel. Then, PCF was obtained after drying, and the reaction mechanism, chemical structures and photographs of PCF are shown in Figure 2.

### 3.2. FT-IR Analysis

**Figure 3:**
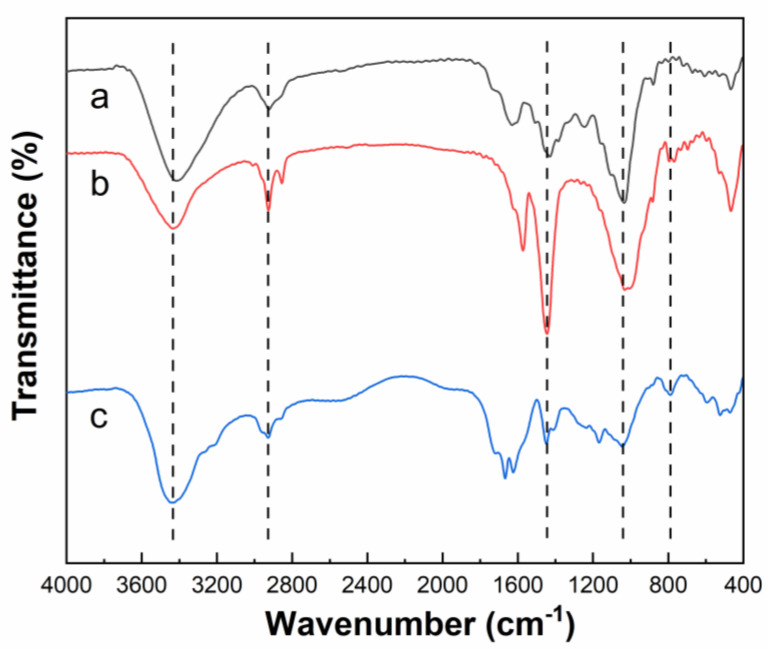
FT-IR spectra of CS (**a**), geopolymer (**b**) and PCF (**c**).

In the spectrum of CS, geopolymer, and PCF (Figure 3), the characteristic absorption peak of cellulose structures in CS (-OH stretching vibration at 3409 cm^−1^ and asymmetric stretching vibration at 2938 cm^−1^) was obviously present and the characteristic absorption peaks of geopolymer (Si-O-Si antisymmetric stretch at 1027 cm^−1^ and bending vibration at 755 cm^−1^ due to the polycondensation of alternating Si-O and Al-O bonds) appeared in PCF. In addition, two absorption peaks at 1723 and 1665 cm^−1^ corresponded to the -C=O stretching vibration and the C-N stretching vibration from urea, respectively. These results demonstrate that the CS has been successfully grafted to polymer chains, and geopolymer and urea have been successfully embedded in the polymer network.

### 3.3. SEM and EDS Analysis

**Figure 4:**
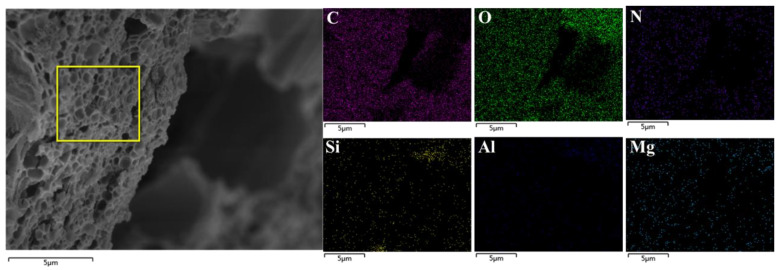
SEM and EDS mapping of PCF.

In Figure 4, PCF shows a porous network structure and rough surface caused by the blend of PAA and geopolymer to form a porous polymer network structure. Meanwhile, the surface elements of PCF were measured by EDS analysis, and the uniform presence of C, N, O, Si, and Al is observed, which is consistent with the loess-based geopolymer, corn straw, and urea. It indicates the composite of the polymer, the geopolymer and urea In PCF.

### 3.4. Compressive Strength Property

**Figure 5:**
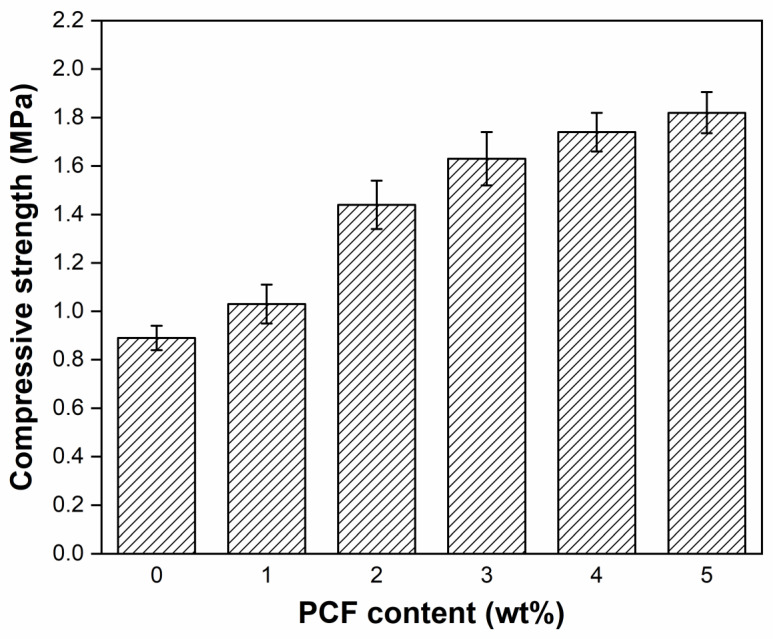
The compressive strength of loess-column samples with different PCF contents.

The compressive strength is an important parameter to evaluate the cohesion and the deformation resistance between soil particles, especially in the loess slopes, where there are large severe landslides and mudslides due to water and soil erosion. To assess the effect of the content of PCF on the loess slope-fixing property, the compressive strength of the loess columns with the different PCF contents (from 1% to 5%, based on the weight of dry loess) was evaluated, and shown in Figure 5. It can be seen that the compressive strength of the soil columns increased with the increase of PCF contents. Compared with the blank group, the compressive strength was increased from 0.89 MPa to 1.82 MPa. It may be due to the swelling of PCF in the loess column sample after absorbing water, which makes the macromolecules chains of polymer in PCF enwrap and entangle the surrounding loess particles and form physical binding between PCF and loess particles. 

### 3.5. Remediation Loess Acidity and Alkalinity by PCF

**Figure 6:**
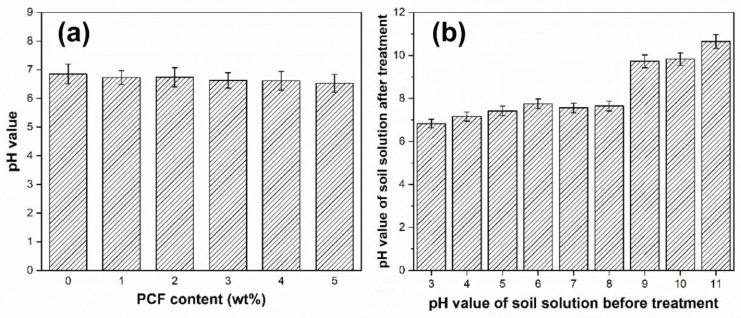
pH value of the loess solution varies with the PCF content (**a**) and the effects of PCF on acidity and alkalinity of the soil solution (**b**).

Figure 6a shows the effects of different content of PCF on the pH value of loess solution. It can be seen that the pH value of loess solution decreases slightly with increasing PCF. Overall, the addition of PCF has little impact on the weakly acidic simulated loess solution (pH = 6.85). 

In addition, in order to study the regulating effect of PCF on different pH values of loess solution, the factors of PCF on the acidity and alkalinity of loess are investigated, as shown in Figure 6b. When the pH value of the simulated loess solution is lower than nine, it can be adjusted to near neutral or weakly acidic after being treated with PCF. When the simulated loess solution pH > 9, PCF had no obvious regulating ability, which indicates that PCF did not interfere with the original alkaline loess environment. It is because PCF has large amounts of –COO^−^ groups, which can react with H_3_O^+^ of the loess solution [28]. Therefore, PCF can regulate the pH value of soil, especially acid soil, and it can be applied in environmental remediation on loess slopes as a kind of amendment. 

### 3.6. Thermal Aging Property

**Figure 7:**
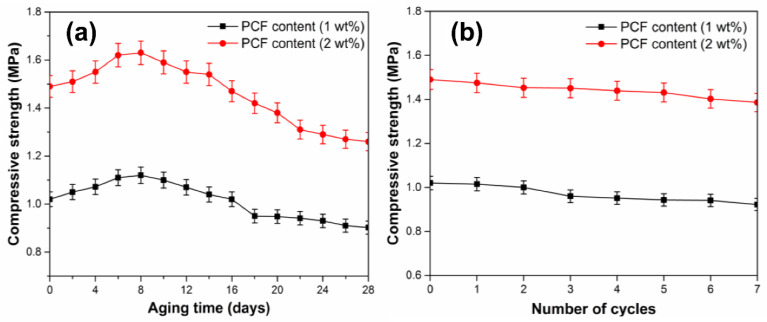
Relationships between time and compressive strength in thermal aging test (**a**) and freeze-thaw aging test (**b**).

The effect of thermal aging on the compressive strength of the loess column samples with 1% and 2% PCF is shown in Figure 7a. It can be observed that the compressive strength of samples increased firstly and then decreased gradually with increasing aging time. It may be because the compressive strength of samples improved with increasing cross-linking density of the PCF in the early stage. However, due to the degradation of PCF under excessive and prolonged heating, the compressive strength decreased slightly in the subsequent test, which indicated that the PCF can maintain stability and excellent thermal stability [29]. 

### 3.7. Freeze-Thaw Resistance Performance

Loess is widely distributed in the northwest of China, where repeated seasonal freeze-thaw cycles damage soil structures [30]. Therefore, the influence of freeze-thaw resistance performance of PCF on loess slope fixation and stability was tested and the results are shown in Figure 7b. It could be seen that the compressive strength of loess column samples with 1 wt% and 2 wt% PCF decreased slightly with increasing freeze-thaw cycles, which may contribute to the destruction on the surface of the loess column samples brought by multiple freeze-thaw cycles. Moreover, the compressive strength of the loess column samples mixed with 1 wt% PCF reduced only 0.1% after seven freeze-thaw cycles, which suggests that the PCF has a good freeze-thaw resistance performance to cope with temperature changes in loess slopes.

### 3.8. Surface Curing Test

**Figure 8:**
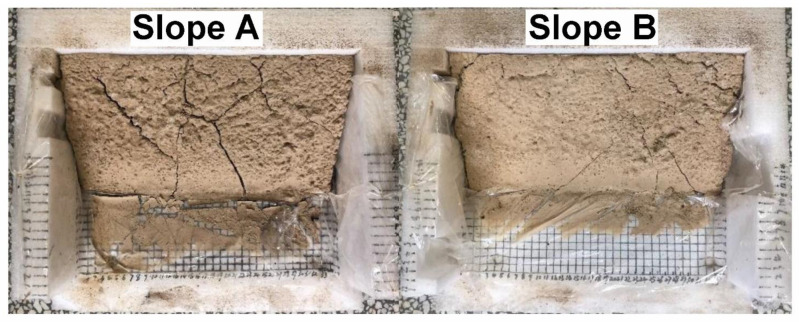
Surface curing images of loess slopes without PCF (**Slope** **A**) and with 1 wt% PCF (**Slope** **B**) after 60 days.

It is well known that surface curing can effectively prevent the slope from runoff. The surface curing of different loess slopes is shown in Figure 8. It can be seen clearly that the loess slope without PCF (Slope A) is uneven and has a lot of large and deep cracks, but the surface treated with PCF (Slope B) is solid and smooth with fewer cracks due to the adhesion and connection of polymer chains in PCF after swelling. In addition, the COAs of different samples in loess slopes were measured and the results are shown in Table 1. The COAs of loess slopes treated with PCF are 91.64% and much higher than the blank group (76.85%), which protects loess slopes against wind and rain erosion. Furthermore, the effects of application methods on the COAs of loess were also studied comparatively. Table 2 shows that the COAs of dry and wet loess by the soil-mixing method are higher than that of the spray-seeding method. Therefore, it can be concluded that the application of PCF could retard evaporation, retain moisture, and effectively prevent soil erosion on loess slopes over a long period of time in arid areas. 

### 3.9. Washing Resistance Property

**Figure 9:**
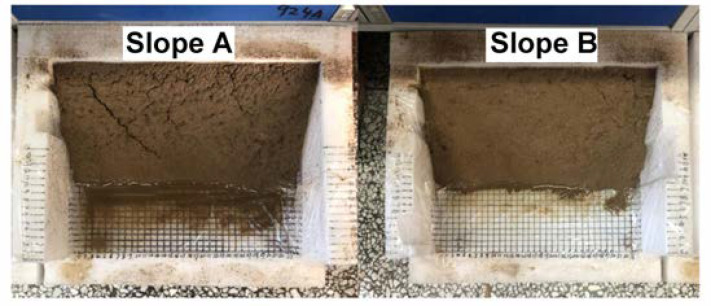
Washing images of loess slopes treated with PCF after spraying with tap water.

The washing resistance property of loess slopes with PCF was investigated. Figure 9 shows that there is no obvious change in the loess slope treated with PCF after spraying with tap water. However, the surface of the loess slope without PCF has some cracks and there is a lot of loess washed downhill from the slope. Meanwhile, the cover area and mass of loess washed down were measured, and the results are shown in Table 3. It can be clearly seen that both the cover area and mass of loess washed down from the slope treated with PCF are significantly lower than that of the slope without PCF. It may be due to the swelling of PCF after absorbing water (the swelling ratio is 181.58 g/g in distilled water) and the enhancement of soil particle coherence after adding long-chain macromolecules, which help prevent soil and water erosion and improve the washing resistance property of loess slope.

### 3.10. Wind Erosion-Resistance Property

**Figure 10:**
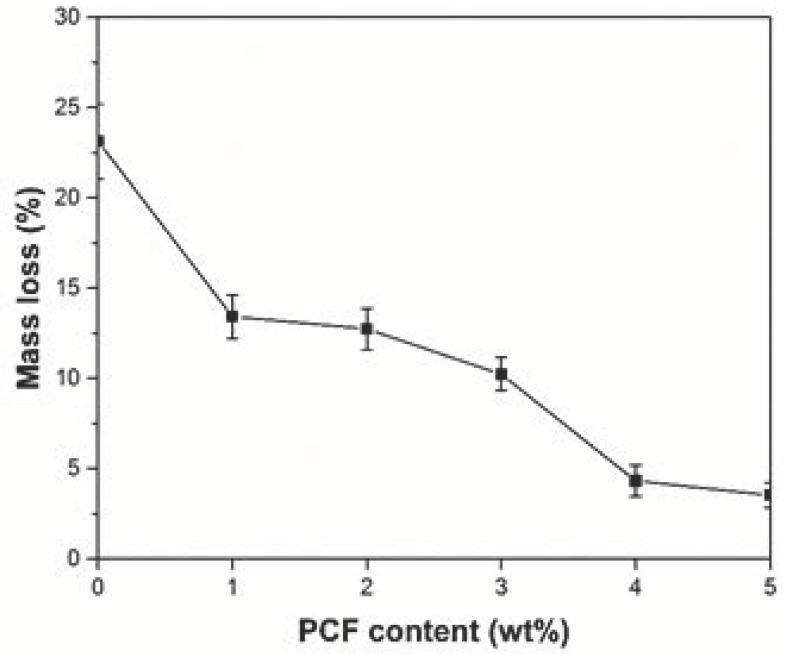
Soil mass loss of loess slope caused by wind erosion with different PCF contents.

Wind erosion-resistance properties have a great influence on PCF application in the arid and semi-arid region of the Loess Plateau, especially for loess slope fixation and stability. Wind erosion-resistance tests were carried out, and the results are shown in Figure 10. With the increase in PCF contents, the mass loss of surface loess in slope reduced from 23.14–3.53%. It shows that applying PCF can improve the wind erosion-resistance performance of loess slopes and reduce the amount of wind erosion of soil.

### 3.11. Degradation Behavior of PCF in Soil

**Figure 11:**
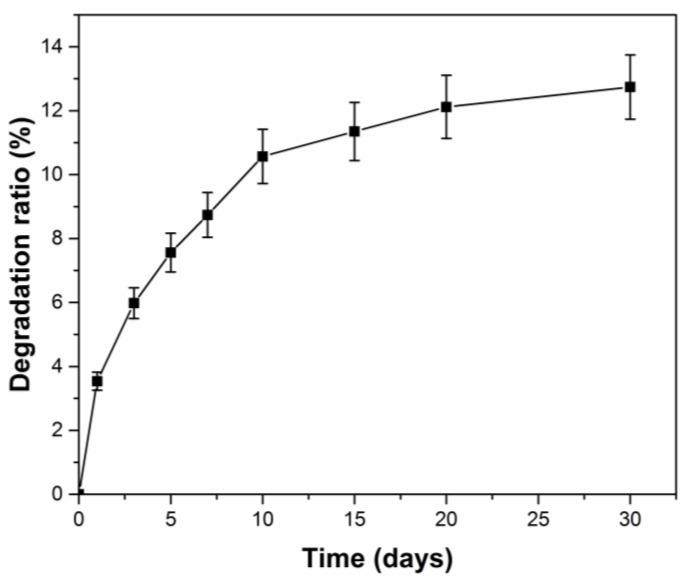
Degradation of PCF in soil during 30 days.

The degradation behavior of PCF was assessed by examining the weight loss in soil at room temperature, as shown in Figure 11. The degradation ratio of PCF gradually increased and reached 12.8% after 30 days. It is observed that PCF shows sustained release behavior and long-term slope protection performance due to the slow and continuous biodegradation in loess.

### 3.12. Influence of PCF on Seed Germination

**Figure 12:**
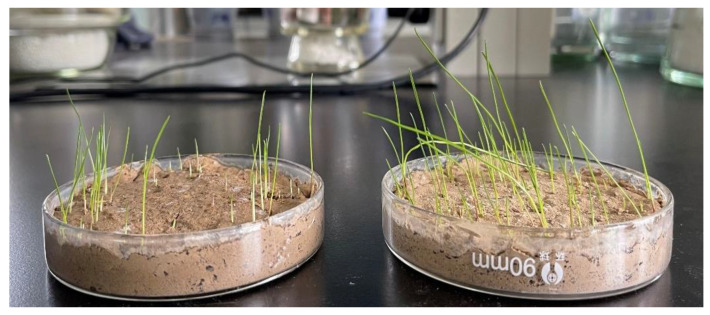
Digital photographs of germination of *Cyperaceae* after treatment with deionized water (**left**) and PCF (**right**) after 7 days.

Figure 12 showed photographs of the germination of *Cyperaceae* seeds after 7 days. From the figure, we can see that there was an influence on the germination of seeds treated with PCF (germination rate: 68%) compared with the blank group (germination rate: 31%), which may be due to the promotion of seed growth by regulating and releasing of water and nitrogen in PCF.

### 3.13. Slope Planting Experiment

**Figure 13:**
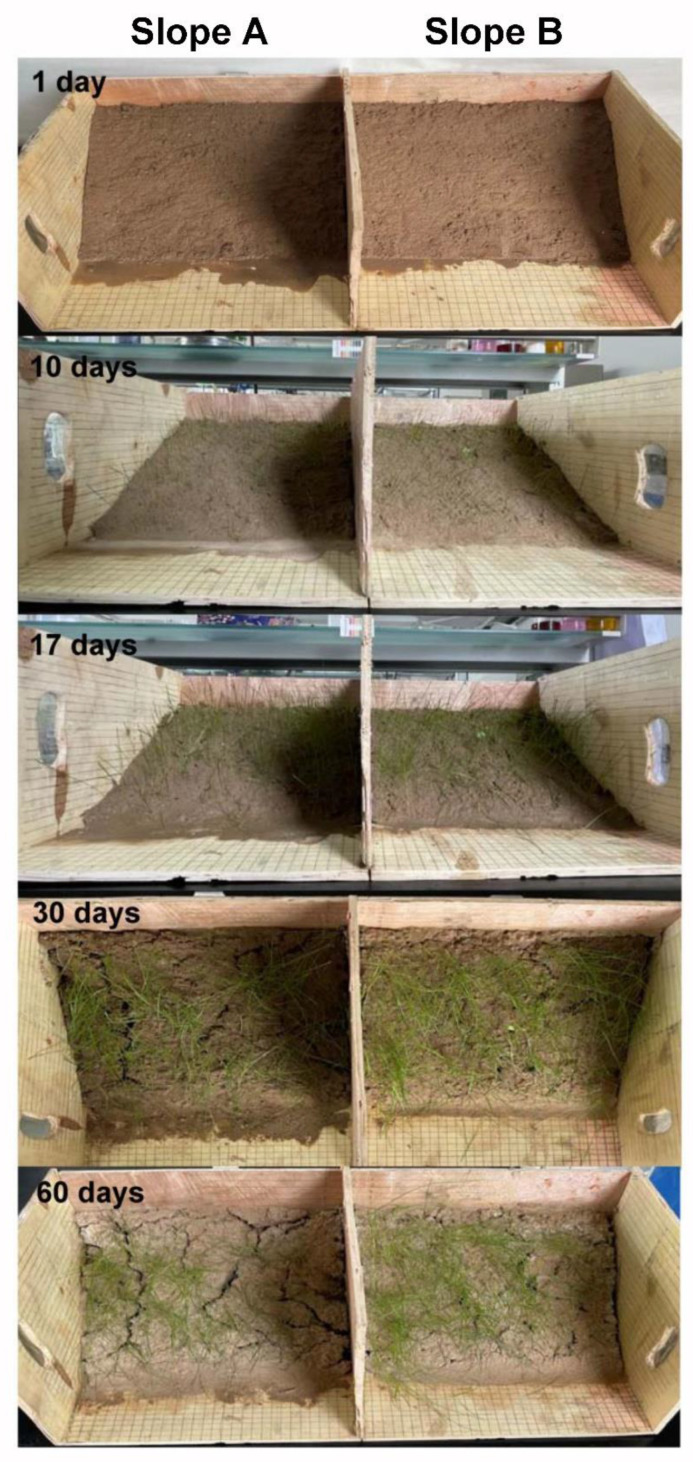
Digital photographs of vegetation (*Cyperaceae*) in loess slopes treated without PCF (**Slope A**) and with 2 wt% PCF (**Slope B**).

**Figure 14:**
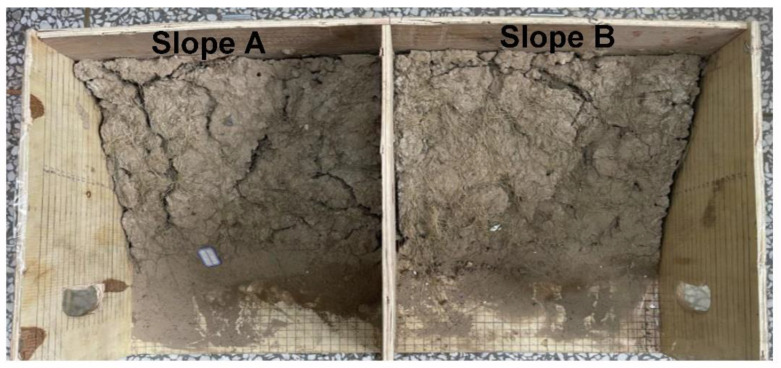
Digital photographs of loess slopes exposed outside without intervention after slope planting experiment.

Slope planting experiments were carried out to determine the applicability of PCF as a nitrogen fertilizer. *Cyperaceae* seeds were exposed to bare loess Slope A and loess Slope B with PCF (B), and their growth is shown in Figure 13. Furthermore, the survival rate of vegetation and the damage to the slope are counted and recorded in Table 4. It can be seen clearly that the *Cyperaceae* involved with PCF show significant improvement in the growth of vegetation compared with the blank group after 60-days of experiments. Meanwhile, compared with Slope B with PCF, there are a lot of long and deep cracks on the surface of Slope A without PCF. To evaluate the long-term effect of slope fixation, the simulated loess slopes for planting experiments after 60 days were placed outdoors without intervention from July to November, and the change in loess slopes was shown in Figure 14. Compared with treatment by PCF, there are more deep cracks on the surface, slight slope sliding, and descent for the blank group. It indicates that PCF not only improves loess slope fixation and stability due to the physical and chemical effects of polymer chains and soil particles but also promotes slope vegetation growth as nitrogen fertilizer to enhance biological protection for loess slopes.

## 4. Conclusions

An eco-friendly polymer composite fertilizer (PCF) based on graft copolymerization of corn straw with acrylic acid and a composite of loess-based geopolymer has been developed, and its loess slope fixation, stability, and erosion control have been evaluated. When the content of PCF was increased from 0% to 5%, the compressive strength was increased from 0.89 MPa to 1.82 MPa. The PCF has not only good heat resistance, washing resistance, freeze-thaw resistance, wind erosion resistance, and surface curing properties, but it also can regulate the pH value of soil to a neutral environment, which will be very useful for loess slope fixation and stability by physical and chemical coefficients. Moreover, PCF can significantly promote the germination rate from 31% to 68% and the survival rate of slope vegetation from 45.2% to 67.7% as a nitrogen fertilizer to enhance biological protection for loess slopes. It is believed that this low-cost and eco-friendly polymer composite fertilizer has significant potential to be applied in slope protection and long-term remediation.

## Figures and Tables

**Table 1:** The COAs of loess slopes.

Samples	COAs
Slope A	76.85%
Slope B	91.64%

**Table 2:** Effects of application methods on the COAs.

Application Method	COAs (Dry Loess)	COAs (Wet Loess)
Spray-seeding method	72.61%	90.63%
Soil-mixing method	80.46%	92.24%

The content of PCF is 1 wt%.

**Table 3:** The cover area and loss mass of loess washed down from the loess slope.

Samples	Bottom Loess Cover Area/cm^2^	Bottom Loess Loss Mass/g
Slope A	238	11.4
Slope B	102	2.7

**Table 4:** The survival rate of vegetation after 60 days of cultivation and the damage to the slopes after 4 months of cultivation outdoors.

Samples	The Survival Rate of Vegetation (%)	Total Crack Length (cm)	Average Crack Width (cm)
Slope A	45.2 ± 3.0	86 ± 5	0.75 ± 0.20
Slope B	67.7 ± 3.0	37 ± 5	0.25 ± 0.20

## Data Availability

The data presented in this study are available on request from the corresponding author.
